# Hyperthermia-Induced In Situ Drug Amorphization by Superparamagnetic Nanoparticles in Oral Dosage Forms

**DOI:** 10.1021/acsami.2c03556

**Published:** 2022-04-22

**Authors:** Shaquib Rahman Ansari, Nele-Johanna Hempel, Shno Asad, Peter Svedlindh, Christel A. S. Bergström, Korbinian Löbmann, Alexandra Teleki

**Affiliations:** † Department of Pharmacy, Science for Life Laboratory, Uppsala University, Uppsala 75123, Sweden; ‡ Department of Pharmacy, University of Copenhagen, Copenhagen 2100, Denmark; § Department of Materials Science and Engineering, Uppsala University, Uppsala 75103, Sweden; ∥ The Swedish Drug Delivery Center, Department of Pharmacy, Uppsala University, Uppsala 75123, Sweden

**Keywords:** superparamagnetic nanoparticles, oral drug delivery, in situ drug amorphization, amorphous solid dispersions, magnetic hyperthermia

## Abstract

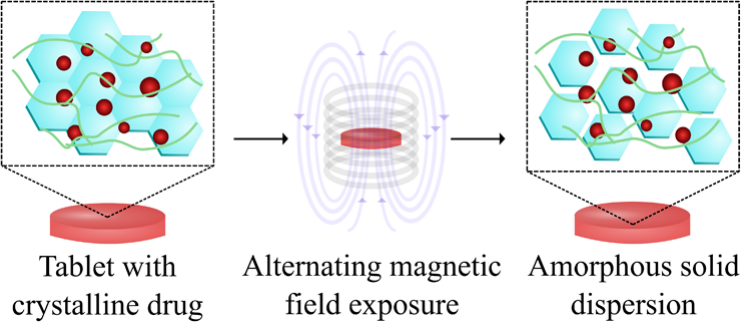

Superparamagnetic iron oxide nanoparticles (SPIONs) generate heat upon exposure to an alternating magnetic field (AMF), which has been studied for hyperthermia treatment and triggered drug release. This study introduces a novel application of magnetic hyperthermia to induce amorphization of a poorly aqueous soluble drug, celecoxib, in situ in tablets for oral administration. Poor aqueous solubility of many drug candidates is a major hurdle in oral drug development. A novel approach to overcome this challenge is in situ amorphization of crystalline drugs. This method facilitates amorphization by molecular dispersion of the drug in a polymeric network inside a tablet, circumventing the physical instability encountered during the manufacturing and storage of conventional amorphous solid dispersions. However, the current shortcomings of this approach include low drug loading, toxicity of excipients, and drug degradation. Here, doped SPIONs produced by flame spray pyrolysis are compacted with polyvinylpyrrolidone and celecoxib and exposed to an AMF in solid state. A design of experiments approach was used to investigate the effects of SPION composition (Zn_0.5_Fe_2.5_O_4_ and Mn_0.5_Fe_2.5_O_4_), doped SPION content (10–20 wt %), drug load (30–50 wt %), and duration of AMF (3–15 min) on the degree of drug amorphization. The degree of amorphization is strongly linked to the maximum tablet temperature achieved during the AMF exposure (*r* = 0.96), which depends on the SPION composition and content in the tablets. Complete amorphization is achieved with 20 wt % Mn_0.5_Fe_2.5_O_4_ and 30 wt % celecoxib in the tablets that reached the maximum temperature of 165.2 °C after 15 min of AMF exposure. Furthermore, manganese ferrite exhibits no toxicity in human intestinal Caco-2 cell lines. The resulting maximum solubility of in situ amorphized celecoxib is 5 times higher than that of crystalline celecoxib in biorelevant intestinal fluid. This demonstrates the promising capability of SPIONs as enabling excipients to magnetically induce amorphization in situ in oral dosage forms.

## Introduction

Poor aqueous drug solubility, and the resulting low bioavailability and potential lack of therapeutic effect, is a major challenge in oral drug delivery. One strategy to increase the solubility and dissolution rate is the conversion of the crystalline drug into its amorphous form.^1^ However, the amorphous forms are thermodynamically unstable and require stabilization to avoid recrystallization during storage or after in vivo administration. The drug is therefore commonly formulated as an amorphous solid dispersion (ASD) in which it is molecularly dispersed in a polymeric network. The polymer in the ASD stabilizes the drug in the solid state and thereby inhibits recrystallization during storage. Further, it may prevent (or delay) drug precipitation upon dissolution and improve solubility. This results in fast dissolution where the subsequent maintenance of supersaturated drug concentrations in vivo can drive absorption from the gastrointestinal tract.^1−3^ Oral dosage forms comprising ASDs have been successfully marketed, for example, VENCLEXTA, which is used to treat diseases such as chronic lymphocytic leukemia and acute myeloid leukemia, contains venetoclax as the active pharmaceutical ingredient and polyvinyl alcohol as the enabling excipient.

However, despite the proven advantages of ASDs, several shortcomings hinder the successful translation of more ASD-based dosage forms to the market.^3,4^ The drug load in stable ASDs is typically low (10–30 wt %) as it is limited by the drug solubility in the polymer at the storage temperature,^2,5^ which in turn means that the size of the dosage form increases when a higher drug dose is required, with negative implications for the patient. Furthermore, many polymers commonly used as ASD excipients, such as polyvinylpyrrolidone (PVP), are hygroscopic. Such excipients increase the molecular mobility in the ASDs due to the plasticizing effect of adsorbed water and hence can induce amorphous–amorphous phase separation and ultimately recrystallization of the drug during storage. Finally, amorphous powders exhibit poor flowability in the pharmaceutical tableting equipment. As a result, ASD manufacturing requires additional processing steps, such as granulation, which increase the complexity and cost of production.

In situ amorphization has been recently introduced to overcome the challenges of drug load, stability, and manufacturing of ASDs.^6^ In situ amorphization can overcome the storage stability issues of ASDs and allow for higher drug loadings (up to 50 wt %) than what is dictated by the thermodynamic solubility limit of the drug in the polymer at the storage temperature as storage times can be kept short.^7,8^ Amorphization and ASD formation take place in the final dosage form prior to ingestion, either directly after tablet manufacturing or before administration to the patient, for example, at hospital pharmacies.^9^ The tablet for in situ amorphization is produced by established pharmaceutical tableting protocols such as direct compaction of a crystalline drug in a polymer mixture. The ASD is then formed in situ by exposing the tablet to a radiation source, for example, microwave^9,10^ or laser.^8^ This is a time- and temperature-dependent process, whereby the radiation increases the tablet temperature and the drug dissolves in the polymer at temperatures above the glass-transition temperature (*T*_g_) of the polymer.^7,11^ Amorphization by microwave radiation requires excipients, such as glycerol or water, in the tablets.^9^ Large amounts (approx. 20 wt %) of enabling excipients have to be added to reach complete amorphization, which hampers the mechanical properties of the tablet. The laser-induced approach uses plasmonic nanoparticles (Ag) as the enabling excipients and only requires as low as 0.1 wt % of them in the tablets.^8^ However, the penetration depth of laser in the tablet is limited and not uniform, making the scale-up difficult. Furthermore, the use of plasmonic nanoparticles may be toxic upon repeated administration to patients due to their dose-dependent toxicity.^12^ Thus, there is a need to identify other effective and safe in situ amorphization excipients.

Superparamagnetic iron oxide nanoparticles (SPIONs) could overcome the shortcomings of current in situ amorphization methods by using magnetic hyperthermia. SPIONs (Fe_3_O_4_ or γ-Fe_2_O_3_) are nontoxic, biocompatible, biodegradable, and efficiently cleared from the body via the iron metabolism pathway.^13^ They are approved by the US Food and Drug Administration as oral magnetic resonance imaging contrast agents (ferumoxsil) and for the treatment of anemia (ferumoxytol) in patients with chronic kidney disease.^14^

SPIONs release heat locally upon exposure to an alternating magnetic field (AMF) due to relaxation losses. The heat dissipation from SPIONs on exposure to an AMF is the result of relaxation of the magnetic moment within the particle (Néel relaxation) or the rotation of the particle itself (Brownian relaxation).^15^ So far, this property of SPIONs, commonly termed magnetic hyperthermia, has been used for the treatment of cancer^16,17^ and triggered drug delivery.^18^ The heat dissipation properties can be enhanced by careful engineering of the SPION properties, for example, with unidirectional growth of nanoparticles, doping with metals, size optimization, and formation of nanocrystal clusters.^19−21^ In particular, spinel crystal nanostructures of, for example, iron oxide doped with Zn^2+^ or Mn^2+^ release more heat than pure iron oxides.^22,23^

**Figure 1:**
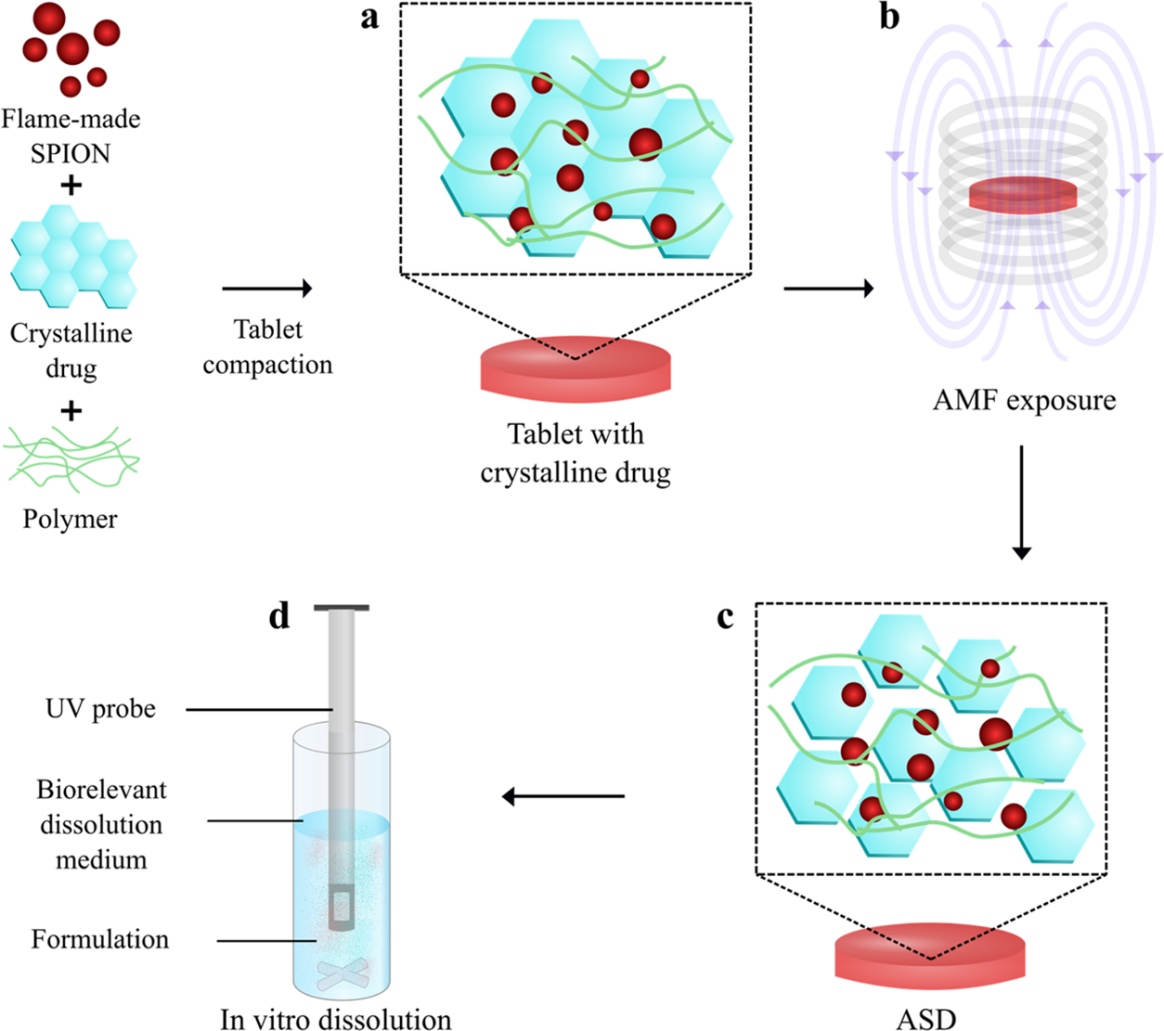
Amorphization by magnetic hyperthermia of a poorly aqueous soluble, crystalline drug in a tablet. (a) Crystalline drug is compacted into tablets with flame-made doped SPIONs and a polymer. (b) Tablets are exposed to an AMF to form an (c) ASD. (d) Performance of the ASD is evaluated by an in vitro dissolution assay.

In this work, we demonstrate for the first time the use of doped SPIONs to induce amorphization of a poorly aqueous soluble crystalline drug (celecoxib) in tablets for oral administration (Figure 1). Celecoxib, a cyclooxygenase-2 inhibitor, widely used to treat osteoarthritis and rheumatoid arthritis,^24^ was chosen as the model drug due to its low aqueous solubility and poor bioavailability. Because of this, high doses are required for oral administration, which causes adverse side effects in the gastrointestinal tract. The undoped and doped SPIONs were produced by flame spray pyrolysis (FSP),^23,25^ a bottom-up nanomanufacturing technique with proven scalability and reproducibility.^26,27^ The structural, morphological, magnetic, and heating properties of the FSP-made nanoparticles were characterized, and their cytotoxicity on human Caco-2 intestinal cells was assessed. A design of experiments (DoE) approach was applied to systematically investigate the effects of nanoparticle and tablet composition, and AMF exposure time, on the degree of drug amorphization in the tablets. Finally, the dissolution behavior of the tablets containing manganese ferrite nanoparticles was evaluated in vitro in biorelevant simulated intestinal fluids. This study thus reports a novel application of SPION-induced magnetic hyperthermia to improve the oral drug delivery of a poorly aqueous soluble drug candidate.

## Results and Discussion

### Nanoparticle Properties

Zinc and manganese ferrites were prepared by FSP and investigated as enabling excipients for in situ amorphization in tablets containing celecoxib. Flame process parameters were set to produce nanoparticles with primary particle sizes in the range of 12–20 nm. This size range has been reported, both experimentally and computationally, to result in maximum heating efficiency during hyperthermia.^28−31^Table S1 summarizes the nanoparticle size, magnetization, and heating efficiency in an AMF of as-produced SPION powders. Pure iron oxide is included for comparison.

**Figure 2:**
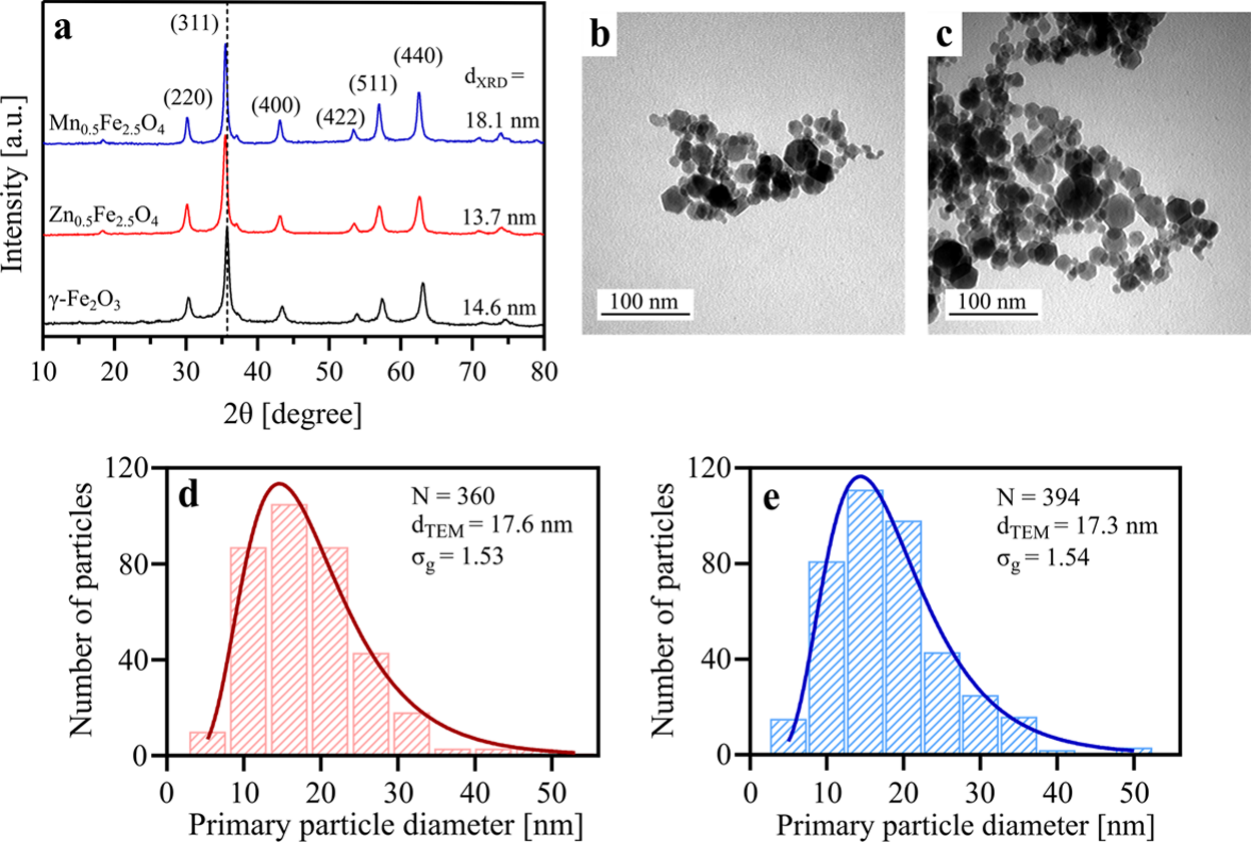
(a) XRD patterns of γ-Fe_2_O_3_ (black), Zn_0.5_Fe_2.5_O_4_ (red), and Mn_0.5_Fe_2.5_O_4_ (blue). The dashed line represents the maghemite (311) peak and visualizes the peak shift for the zinc and manganese ferrites. Representative TEM images and the corresponding primary particle size distributions of (b,d) Zn_0.5_Fe_2.5_O_4_ and (c,e) Mn_0.5_Fe_2.5_O_4_. The solid lines (d,e) represent the log-normal size distribution fit.

Figure 2a shows the X-ray diffraction (XRD) patterns of pure γ-Fe_2_O_3_ and SPION doped with Zn^2+^ and Mn^2+^ to form the ferrites, Zn_0.5_Fe_2.5_O_4_ and Mn_0.5_Fe_2.5_O_4_. The diffractograms correspond to spinel cubic structures, exhibiting six prominent peaks originating from the (220), (311), (400), (422), (511), and (440) crystallographic planes.^32,33^ Pure iron oxide forms γ-Fe_2_O_3_ (maghemite), in agreement with previous SPION synthesis in oxygen-rich flames.^34^ Spinel ferrite structures were formed by the addition of Zn^2+^ or Mn^2+^, as evidenced by a slight shift of diffraction peaks toward lower angles [shown in Figure 2a as the dashed line at the (311) plane].^25^ The peak shift is accompanied with a lattice expansion (Table S1), indicating the successful incorporation of Zn^2+^ and Mn^2+^ ions into the γ-Fe_2_O_3_ crystal lattice.^23,35^ Furthermore, no diffraction peaks corresponding to pure zinc oxide or manganese oxide were observed. The average crystallite size (*d*_XRD_) was about 14 nm for both γ-Fe_2_O_3_ and Zn_0.5_Fe_2.5_O_4_ and 18 nm for Mn_0.5_Fe_2.5_O_4_ (Figure 2a). The larger ionic radius of Mn^2+^ (0.81 Å) compared to Fe^3+^ (0.64 Å) and Zn^2+^ (0.74 Å) at the tetrahedral site might explain the increase in crystal size for manganese ferrite.^36,37^

Figure 2b,c shows the representative transmission electron microscopy (TEM) images of Zn_0.5_Fe_2.5_O_4_ and Mn_0.5_Fe_2.5_O_4_ nanoparticles. The nanoparticles are polyhedrons with a narrow primary particle size distribution (Figure 2d,e), in excellent agreement with the literature.^25,34,38^ The primary particle sizes (*d*_TEM_) determined from the TEM analysis by log-normal distribution fitting were 17.6 and 17.3 nm for zinc and manganese ferrites, respectively. These are in good agreement with *d*_XRD_ and thus suggest primarily monocrystalline particles. The *d*_TEM_ value also falls within the targeted optimal range of SPION particle size (12–20 nm) for high heating rates.^28−30^ The geometric standard deviation (σ_g_) was 1.53 and 1.54 for Zn_0.5_Fe_2.5_O_4_ and Mn_0.5_Fe_2.5_O_4_, respectively, and thus corresponds to the theoretical self-preserving size distribution that is typically attained during flame synthesis.^39^

### Magnetic Properties and Heating Efficiency

**Figure 3:**
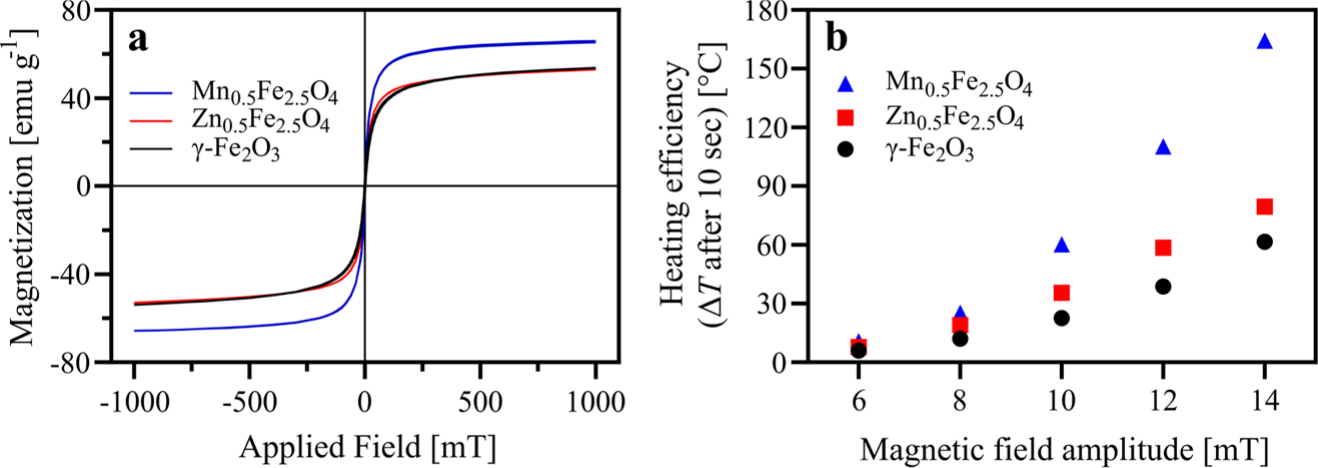
(a) Magnetization curves at 300 K for undoped and doped SPIONs (γ-Fe_2_O_3_: black; Zn_0.5_Fe_2.5_O_4_: red; and Mn_0.5_Fe_2.5_O_4_: blue). (b) Heating efficiency of the undoped and doped SPION powders at 588.5 kHz and different magnetic field amplitudes for γ-Fe_2_O_3_ (black circles), Zn_0.5_Fe_2.5_O_4_ (red squares), and Mn_0.5_Fe_2.5_O_4_ (blue triangles).

Figure 3a shows the magnetization of flame-made undoped and doped SPIONs. The nanoparticles exhibit nearly zero hysteresis, confirming their superparamagnetism. At the maximum field of 1000 mT, nearly all particles reached their saturation magnetization. Bulk maghemite has a saturation magnetization of 80 emu g^–1^, which is considerably higher than the 53.7 emu g^–1^ measured for γ-Fe_2_O_3_ here (Table S1). This can be attributed to the small particle size that increases the thickness and the mass fraction of the surface spin-disordered layer, also called the “magnetically dead layer”.^40^ Mn_0.5_Fe_2.5_O_4_ exhibits the highest saturation magnetization (66.7 emu g^–1^) of the flame-made nanoparticles here, in agreement with the literature.^33^ The dopant occupies the tetrahedral or octahedral sites in the structure of γ-Fe_2_O_3_, which affects the magnetocrystalline anisotropy and increases the overall magnetic moment of the unit cell, thereby also affecting the saturation magnetization of the SPIONs.^41^ Surprisingly, the incorporation of Zn^2+^ in γ-Fe_2_O_3_ did not enhance the saturation magnetization in contrast to previous reports.^32,42^ This could be due to the smaller size of zinc ferrite compared to manganese ferrite and hence the increased relative volume contribution of the magnetically dead layer to the total particle volume for the former. Moreover, the high substitution of Fe^3+^ ions by Zn^2+^ ions at the tetrahedral site also weakens the exchange coupling between the tetrahedral and octahedral sites, thus decreasing the total magnetic moment of the unit cell.^42^

Figure 3b shows the heating efficiency (represented as the temperature increase Δ*T* after 10 s) of the nanoparticle powders in an AMF. The heating efficiency was directly measured on the bulk powders (in contrast to how particle suspensions are typically measured) as they were used here in their dry state in the final tablet formulations; hence, the heat dissipation primarily originates from the Néel relaxation of the undoped and doped SPIONs. Zinc ferrite, and especially manganese ferrite, generated significantly higher temperatures than γ-Fe_2_O_3_, in agreement with previous reports.^22,23^ The heating efficiency of SPIONs is often correlated with the saturation magnetization,^32^ and this was especially obvious for Mn_0.5_Fe_2.5_O_4_ (Figure 3a). Furthermore, the heating efficiency increased with the increasing magnetic field amplitude, also in agreement with previous studies.^32,43^ Overall, these results demonstrate the superior heating efficiency of Zn and Mn ferrites compared to γ-Fe_2_O_3_. We therefore investigated these two ferrites further for the magnetic hyperthermia-induced in situ amorphization.

### Magnetic Hyperthermia-Induced In Situ Drug Amorphization in Tablets

**Figure 4:**
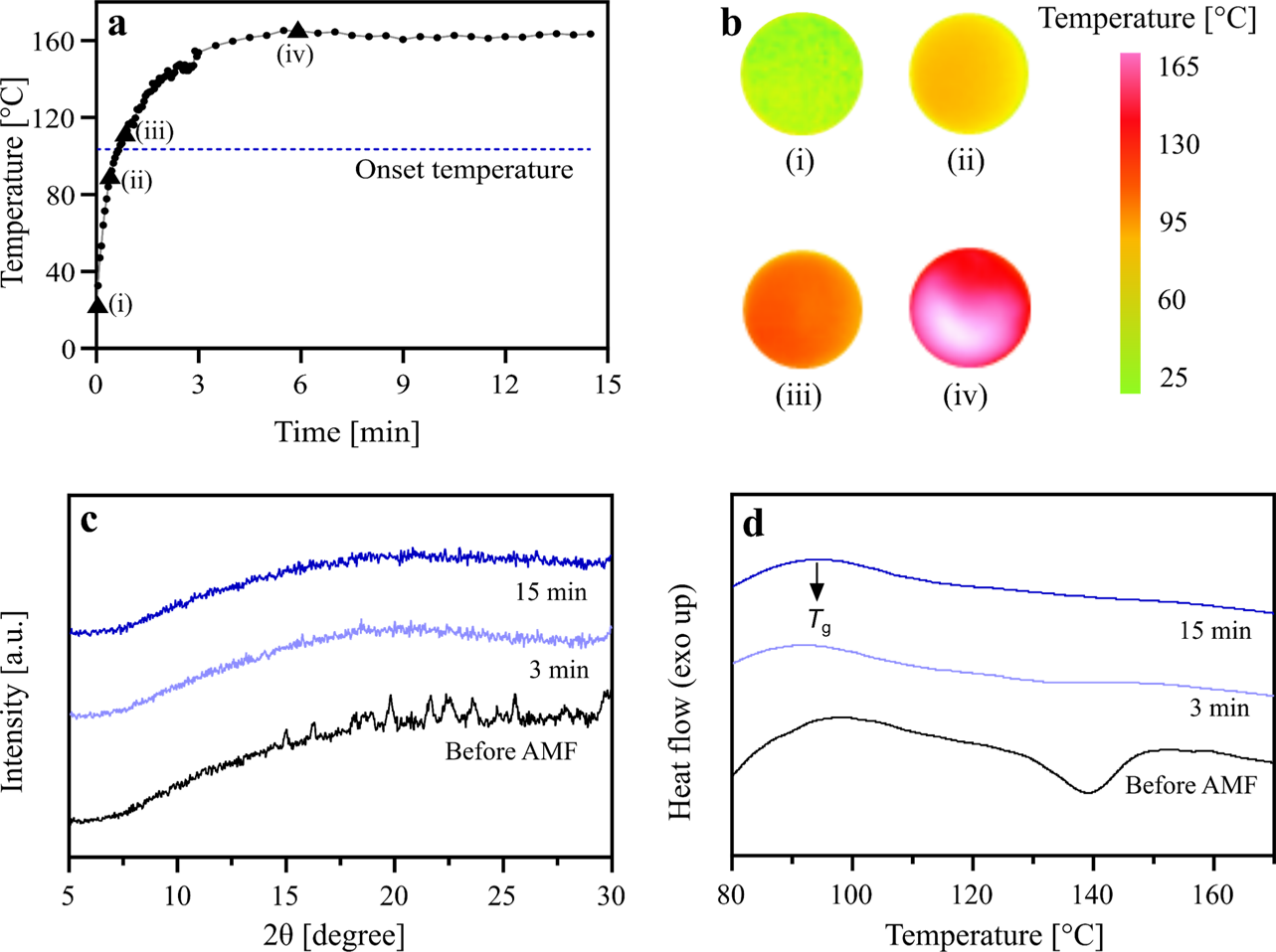
(a) Tablet temperature as a function of AMF exposure time for a tablet containing 30 wt % celecoxib and 20 wt % Mn_0.5_Fe_2.5_O_4_ (tablet 26, Table S3). (b) IR images captured at four time points [(i) 0, (ii) 0.5, (iii) 1, and (iv) 6 min]. The color scale indicates the temperature of the tablet. (c) XRD diffractograms and (d) DSC thermograms of tablets containing 30 wt % celecoxib and 20 wt % Mn_0.5_Fe_2.5_O_4_ before and after 3 and 15 min of AMF exposure time.

Tablets were prepared based on the formulation design space (Table S2) constructed using DoE to investigate the factors for magnetic hyperthermia-induced drug amorphization. Table S3 summarizes the tablet compositions, process conditions, and the observed degree of amorphization. The tablet containing 30 wt % celecoxib and 20 wt % Mn_0.5_Fe_2.5_O_4_ exposed to AMF in solid state for 15 min (tablet 26, Table S3) showed the maximum amorphous content induced by in situ amorphization and is thus presented here in detail (Figure 4). Figure 4a shows the temperature profile of this tablet, and the thermal images recorded with an IR camera at four time points are shown in Figure 4b. The tablet surface temperature rapidly increased from room temperature to 154 °C within the first 3 min of AMF exposure. The tablet temperature further increased to a maximum (*T*_max_) of 165 °C after 6 min, which was maintained for the remaining time of AMF exposure. As the doped SPIONs are homogeneously distributed in the matrix, hyperthermia results in uniform heating of the entire tablet, as shown here by the monocolored thermal images (Figure 4b). The uniform heating ensures complete drug amorphization in the tablets. This is a clear advantage of magnetic hyperthermia-induced amorphization over the previously reported laser radiation approach. The latter can only be applied to thin tablets due to the limited penetration depth of the laser.^8^

Some of the tablets showed deformation after AMF exposure due to water evaporation and increased polymer mobility above the *T*_g_ value of PVP (here, *T*_g_ = 80–85 °C). A significant decrease in viscosity of the polymer has been observed at approximately 15–25 °C above the *T*_g_ value of the polymer,^44,45^ enabling the drug dissolution and amorphization process. Here, the onset temperature for drug amorphization was determined by differential scanning calorimetry (DSC) and was found to be 103.6, 117.7, and 117.6 °C for the drug load of 30, 40, and 50 wt % respectively [*T*_g_ + (15–25) °C depending on the wt % of celecoxib]. At temperatures > *T*_g_, the polymer viscosity decreases, leading to a drastic increase of molecular mobility in the network. Thus, the drug dissolves easily into the polymer,^44^ and in situ amorphization is initiated. The AMF-exposed tablet in Figure 4a significantly exceeded the onset temperature required for the amorphization of celecoxib and was maintained above that temperature for an adequate time, thus ensuring an efficient in situ amorphization by hyperthermia.

The degree of amorphization after the AMF exposure of the tablets was quantified by XRD and DSC. Figure 4c shows the XRD diffractograms of tablet 26 as prepared and after 3 and 15 min of AMF exposure. The as-prepared tablet displays the characteristic diffraction peaks of crystalline celecoxib. The intensity of the crystalline peaks diminished already after 3 min of AMF exposure. Complete amorphization was achieved after 15 min, as indicated by the “halo” characteristic of an amorphous material. Similarly, the depressed melting endotherm of crystalline celecoxib in the presence of PVP at about 140 °C in the as-prepared tablets was no longer detected by DSC after AMF exposure (Figure 4d). The successful amorphization is evident in the DSC thermograms from a single glass transition (*T*_g_) of the polymer after in situ amorphization (Figure 4d). Furthermore, no degradation products of celecoxib were observed in AMF-exposed tablets by HPLC-UV analysis (data not shown), in agreement with the laser-induced in situ amorphization of celecoxib in PVP.^8^ The long-term stability of amorphous-based formulations is typically classified according to the glass stability of the organic compound and strongly depends on the physicochemical properties of the drug and storage conditions.^46^ Thus, long-term stability needs to be assessed for each in situ amorphized drug compound individually, and celecoxib serves only as a model compound here.

### Modeling of the Maximum Tablet Temperature and Degree of Drug Amorphization

A multiple linear regression model (Table 1) was generated from the tablet data (Table S3) to investigate the effects of process parameters on drug amorphization. Response modeling resulted in high *R*^2^ and *Q*^2^ values for the degree of drug amorphization (*R*^2^ = 0.917, *Q*^2^ = 0.842) and *T*_max_ (*R*^2^ = 0.906, *Q*^2^ = 0.865). Moreover, the predicted and observed values showed strong correlation (Figure S1). It should be noted that an experiment outside the action limit of ±4 standard deviations was identified as an outlier based on the deleted studentized residuals and removed from the final regression model.

**Table 1:** Adjusted Models for the Degree of Drug Amorphization and Maximum Tablet Temperature (*T*_max_)

	degree of drug amorphization		*T*_max_	
model terms	coefficient	*p*-value	coefficient	*p*-value
composition (Mn_0.5_Fe_2.5_O_4_)	**21.26**^a^	**≤0.0001**^a^	19.42	≤0.0001
composition (Zn_0.5_Fe_2.5_O_4_)	**–21.26**^a^	**≤0.0001**^a^	–19.42	≤0.0001
doped SPION content	20.13	≤0.0001	**22.23**^a^	**≤0.0001**^a^
drug load	–6.109	≤0.01		
AMF time	*2.459*^b^	*0.2808*^b^	*2.928*^b^	*0.1788*^b^
AMF time × AMF time	*–5.335*^b^	*0.1313*^b^		
doped SPION content × composition	8.730	≤0.001	5.240	≤0.05
doped SPION content × AMF time	*4.514*^b^	*0.07447*^b^		

a **Bold**: factors with the most influence on the given response.

b *Italics*: nonsignificant parameters retained to conserve either the hierarchy or the predictability of the model.

Doped SPION content in the tablets had the strongest positive influence on tablet temperature (Table 1). For all experiments, the tablet temperature initially increased rapidly with the exposure time until it plateaued (Table S3), and *T*_max_ was then maintained until the end of AMF exposure, which is also shown in tablet 26 (Figure 4a). Mn ferrite showed a much stronger influence on tablet temperature than Zn ferrite. Furthermore, *T*_max_ showed a strong linear relationship to the extent of celecoxib amorphization (*r* = 0.96, Figure S3).

**Figure 5:**
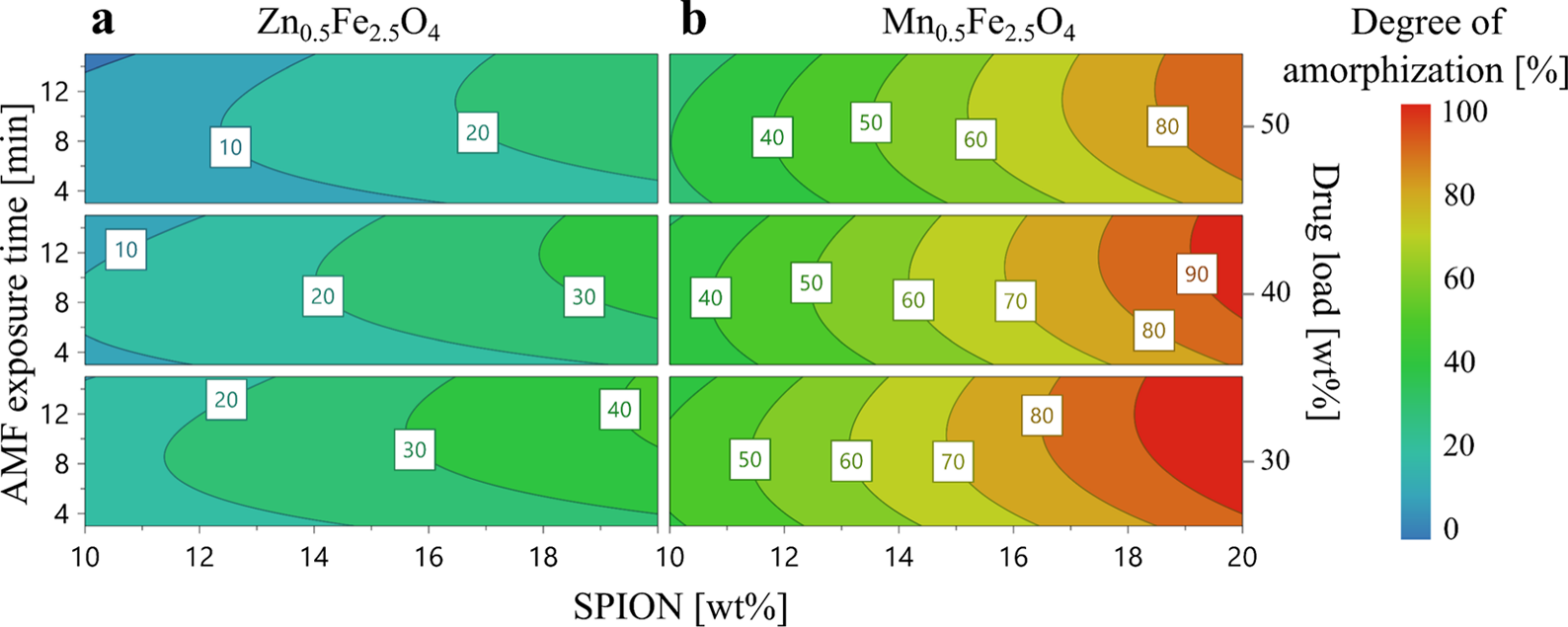
Contour plots showing the degree of celecoxib amorphization for tablets containing (a) Zn_0.5_Fe_2.5_O_4_ and (b) Mn_0.5_Fe_2.5_O_4_ as a function of AMF exposure time, doped-SPION content, and drug load. The color scale indicates the degree of celecoxib amorphization, as measured by DSC.=

SPION composition had the strongest influence on the degree of drug amorphization (Table 1). The contour plots for Zn_0.5_Fe_2.5_O_4_ and Mn_0.5_Fe_2.5_O_4_ (Figure 5) show the extent of drug amorphization as a function of AMF exposure time, drug load, and doped SPION content. Red areas in the contour plot illustrate process conditions that achieved >90% degree of amorphization. Mn_0.5_Fe_2.5_O_4_ was more effective than Zn_0.5_Fe_2.5_O_4_ in the formation of completely amorphous ASDs. This can be attributed to the better heating efficiency of Mn_0.5_Fe_2.5_O_4_ (Figure 3b), resulting in higher tablet temperatures and faster dissolution of celecoxib into the PVP network. The doped SPION content also positively affected the degree of drug amorphization with no significant quadratic term, indicating a linear effect of this parameter in the entire design space. However, the doped SPION content showed a significant interaction term with the SPION composition. In contrast, the tablet drug load had a negative influence on drug amorphization.

Thus, the tablets with 50 wt % drug load showed a lower degree of amorphization compared to that with 30 wt % at similar exposure times and temperatures. This follows the Noyes–Whitney equation^47^ that predicts that for a higher drug load, a higher *T*_max_ can result in complete dissolution in a solvent, here the polymer. Higher temperatures increase the saturation solubility of the drug in the polymer, lower the viscosity of the continuous (polymer) phase, and increase the diffusion coefficient according to the Stokes–Einstein equation. This in turn results in a faster dissolution rate. In fact, for tablets 27 and 28 with a high doped SPION content of 20 wt % (Table S3), celecoxib was not completely amorphized, even though the temperatures reached above the onset temperature of dissolution. This could have been a result of the high drug load (50 wt %) that might require longer exposure times (>15 min) than that applied here to result in complete drug amorphization, as dissolution is a time- and temperature-dependent process. It should be noted that at high drug loads, the drug becomes the bulk of the tablet, resulting in incomplete dissolution of the drug into the polymer also due to insufficient polymer content.

The AMF exposure time was not a significant factor for drug amorphization in the selected design space; however, the model term for the duration of AMF was retained to improve the predictability of the model. The contour plot for tablets containing Zn ferrite (Figure 5a) indicates that the tablets that failed to reach the onset temperature (Table S3) show minimal amorphization despite prolonged AMF exposure. Thus, a minimum AMF exposure time of 3 min was selected to ensure at least some degree of drug amorphization. As a result, the AMF exposure time is a nonsignificant factor in the current model. In future studies, the effect of time on hyperthermia-induced drug amorphization could be investigated by increasing the range and interval of the design. As previously discussed, the heating efficiency of SPIONs depend strongly on the operating AMF amplitude. The maximum AMF amplitude used in this study was confined due to the instrument limitations; however, using higher AMF amplitude could potentially decrease the time required for AMF exposure and the doped SPION content required to achieve complete amorphization.

### Nanoparticle Cytotoxicity on Caco-2 Cells

**Figure 6:**
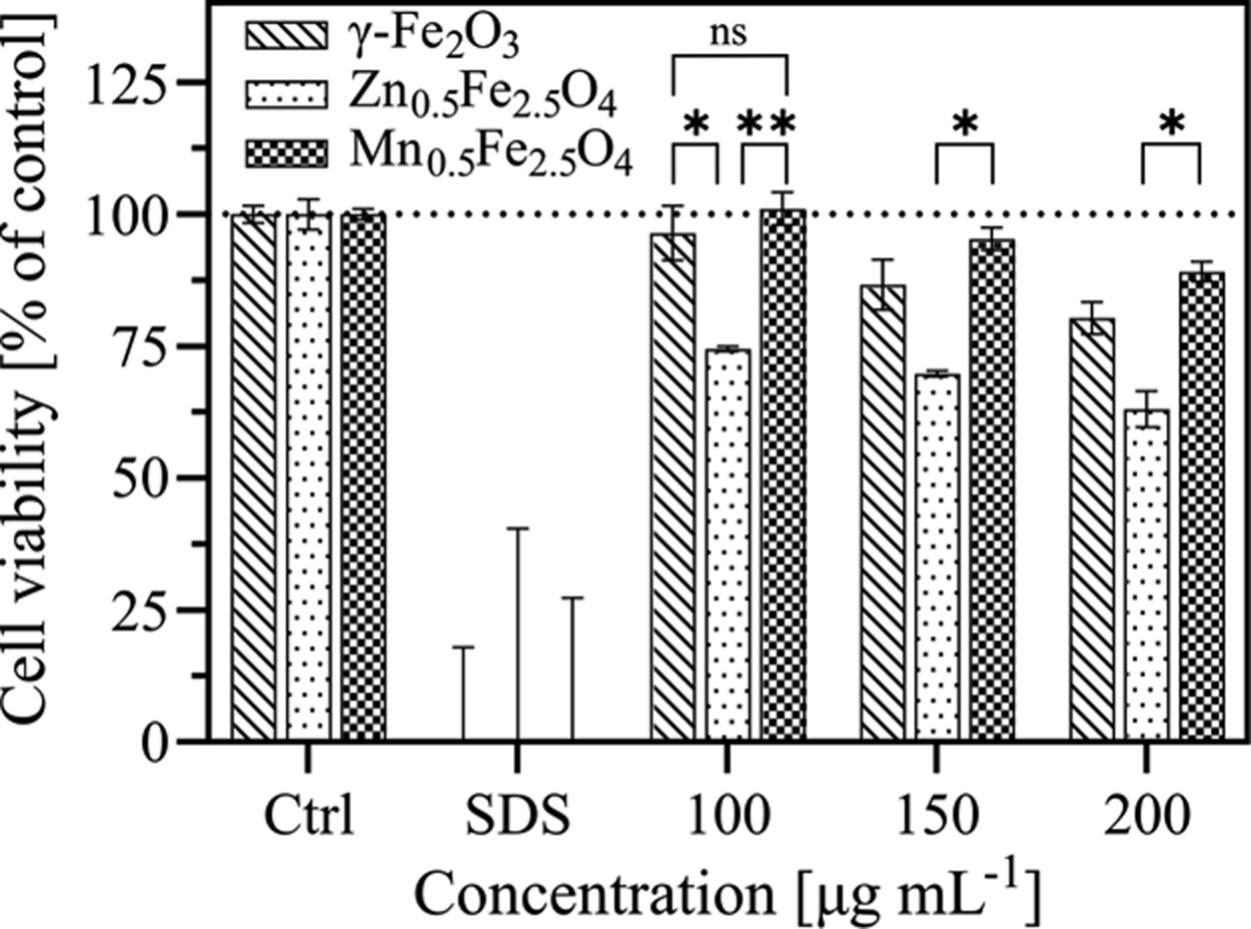
Cell viability of nondifferentiated Caco-2 cells after exposure to γ-Fe_2_O_3_, Zn_0.5_Fe_2.5_O_4_, and Mn_0.5_Fe_2.5_O_4_ at different particle concentrations (100, 150, and 200 μg mL^–1^) for 24 h. Cell viability was determined using the CellTiter-Glo luminescent cell viability assay and calculated as a percentage of the control (set to 100%). A positive control (SDS) is also shown. All data are expressed as mean ± SD. *p* < 0.1234 (ns), 0.0332 (*), 0.0021 (**), 0.0002 (***), and 0.0001 (****).

The most effective hyperthermia-induced drug amorphization was achieved using the Mn_0.5_Fe_2.5_O_4_ nanoparticles (Figure 5b). However, the final choice of an enabling excipient for in situ drug amorphization must also be guided by the potential toxicity of the nanomaterial. Figure 6 shows the dose-dependent viability of human intestinal Caco-2 cells following 24 h of exposure to the undoped and doped SPIONs. Cell viability after nanoparticle exposure is highly dependent on the cell line and differentiation state, exposure time and concentration, and physicochemical properties of the nanoparticles. Here, cell viability was assessed using nondifferentiated cells, which are more sensitive than differentiated ones.^48^ In other words, our model is highly sensitive compared to the in vivo condition and better able to detect any toxic effects exhibited by the flame-made undoped and doped SPIONs. The cell viability was not affected by γ-Fe_2_O_3_ and Mn_0.5_Fe_2.5_O_4_ exposure at 100 μg mL^–1^ (corresponding to an oral dose of 25 mg nanoparticles ingested with a 250 mL glass of water) and was only slightly decreased at higher concentrations. This declining trend of cell viability with increasing particle concentration (Figure 6) suggests a dose-dependent toxicity for all particles. The minor adverse effect of iron oxide nanoparticles on Caco-2 cell viability has also been reported previously.^49^ In contrast, Zn_0.5_Fe_2.5_O_4_ dramatically reduced cell viability to 75% compared to the control already after exposure to 100 μg mL^–1^ and further to 63% at 200 μg mL^–1^. Mn_0.5_Fe_2.5_O_4_, on the other hand, shows significantly higher cell viability than zinc ferrite. Previous studies have also reported a relatively higher cytotoxicity of zinc ferrite compared to manganese ferrite, which can be attributed to the oxidative stress induced by the generation of reactive oxygen species by the former.^22,50^ Overall, the high heating efficiency and low cytotoxicity of the Mn_0.5_Fe_2.5_O_4_ nanoparticles suggest that they are efficient as enabling excipients for hyperthermia-induced in situ amorphization.

### In Vitro Dissolution Studies

**Figure 7:**
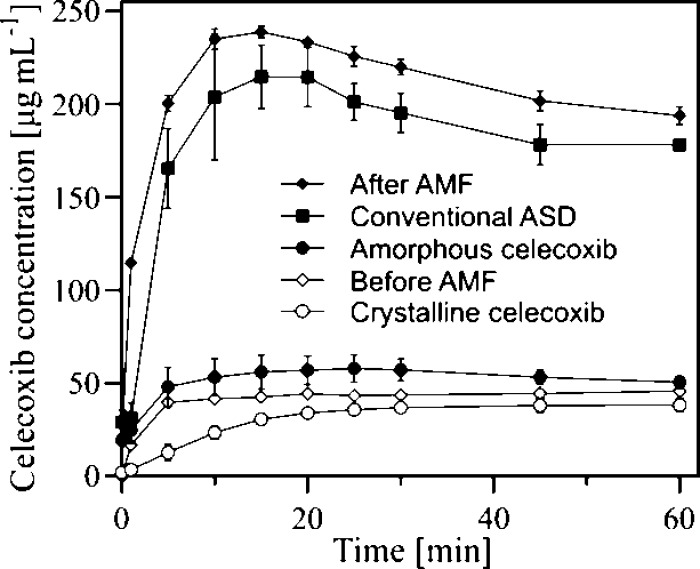
In vitro dissolution profiles of tablets at a dose corresponding to 400 μg mL^–1^ of celecoxib in FaSSIF. Crystalline celecoxib (open circles); amorphous celecoxib (filled circles); conventional ASD of celecoxib (30 wt %) in PVP (filled squares); tablet containing Mn_0.5_Fe_2.5_O_4_ (20 wt %) and celecoxib (30 wt %) in PVP before AMF exposure (open diamonds); and after AMF exposure of 15 min (filled diamonds). Values represent mean celecoxib concentrations ± SD, *n* = 3 (*n* = 2 for tablet containing Mn ferrite after AMF exposure).

In vitro dissolution assays of celecoxib ASDs in biorelevant intestinal media under nonsink conditions have previously shown good correlation with their in vivo performance.^51^ We therefore used them to benchmark the in situ amorphized ASD. Figure 7 shows the in vitro dissolution profiles of crystalline celecoxib, amorphous celecoxib, conventional ASD (prepared by melt quenching), and tablets containing Mn_0.5_Fe_2.5_O_4_, before and after magnetic hyperthermia-induced amorphization. The conventional ASD showed a considerably higher dissolution rate and maximum drug concentration (*C*_max_) than pure crystalline or amorphous celecoxib. This is in good agreement with previous studies demonstrating the superior dissolution characteristics of ASDs compared to pure compounds.^52^ The dissolution profile of ASDs are mainly controlled by the generation and stabilization of a supersaturated solution;^53^ this is commonly referred to as the “spring and parachute effect” and suggests that the performance of an ASD is governed by the dissolution rate and crystallization inhibition of the polymer. PVP maintains the balance between the dissolution rate enhancement and precipitation inhibition. The tablets containing Mn ferrites achieved a similar dissolution profile after AMF exposure as the conventional ASD, reaching *C*_max_ of 239 μg mL^–1^ in 15 min. This concentration was significantly higher than *C*_max_ of conventional ASD (215 μg mL^–1^) and approximately 5 times higher than the *C*_max_ value of the doped SPION-containing tablets before AMF exposure (46 μg mL^–1^). At the end of the sampling period (6 h), the AMF-exposed tablets maintained a higher solubility compared to the conventional ASD and the crystalline celecoxib (Figure S4). The crystalline nanoparticles present in the formulation could act as crystalline nuclei and trigger re-crystallization; however, Mn ferrites did not trigger re-crystallization, thus further affirming their suitability as an in situ amorphization-enabling excipient. Overall, this confirms that the novel magnetic hyperthermia-induced amorphization produces ASDs with the same performance as conventional ASDs in terms of their dissolution behavior.

## Conclusions

This work demonstrates the ability of doped SPIONs to induce in situ amorphization in tablets intended for oral administration. Upon exposure to an AMF, elevated temperatures were achieved in tablets containing doped SPIONs, leading to high amorphization and improved in vitro dissolution. The degree of amorphization was strongly linked to the maximum tablet temperature, which depended significantly on the SPION composition, doped SPION content, and the drug load. Complete amorphization and high apparent solubility were achieved in tablets with 20 wt % Mn_0.5_Fe_2.5_O_4_ and 30 wt % celecoxib after 15 min of AMF exposure. Furthermore, unlike laser- and microwave-induced amorphization, these tablets are homogeneously exposed to the AMF and use safer excipients that are less likely to cause drug degradation. The in situ amorphization can be fine-tuned according to the obtained DoE model, and the method can be widely applied to ASDs with other polymer–drug compositions. The use of a biocompatible excipient produced by a scalable synthesis technique, along with the high drug load in ASDs produced by in situ amorphization, makes the developed method a promising tool for a broader use of ASDs as enabling formulations in oral drug delivery.

## Experimental Section

### Particle Synthesis

Zinc and manganese ferrites (Zn_0.5_Fe_2.5_O_4_ and Mn_0.5_Fe_2.5_O_4_) were synthesized by FSP.^27^ Liquid precursor solutions were prepared by dissolving iron(III) nitrate nonahydrate (purity 98%; Sigma-Aldrich, Sweden) and either zinc nitrate hexahydrate (purity 98%; Sigma-Aldrich) or manganese(II) nitrate tetrahydrate (purity 97%; Sigma-Aldrich) in a solvent mixture (1:1) of 2-ethylhexanoic acid (99%; Sigma-Aldrich) and ethanol (>99.7%, HPLC grade; VWR, Belgium) to obtain a total metal concentration of 0.7 M. Reference pure iron oxide (γ-Fe_2_O_3_) was produced from a 0.7 M iron(III) nitrate nonahydrate precursor solution.

The precursor solutions were stirred for at least 1 h at room temperature. Subsequently, the precursor was fed at 6 mL min^–1^ and dispersed using 3 L min^–1^ O_2_ (>99.5%, Linde AGA Gas AB, Sweden) at a constant pressure (1.6 bar). The flame was ignited by a premixed supporting flame of CH_4_ and O_2_ (>99.5%, Linde AGA Gas AB) at flow rates of 1.5 and 3.2 L min^–1^, respectively. A 5 L min^–1^ O_2_ sheath gas was fed through the outermost sinter metal plate of the FSP burner. Gas flow rates were controlled with calibrated mass flow controllers (Bronkhorst, the Netherlands). The particles were collected on a glass fiber filter (Albert LabScience, Germany) with the aid of a Mink MM 1144 BV vacuum pump (Busch, Sweden).

### Particle Characterization

The specific surface area was determined by nitrogen adsorption (Brunauer–Emmett–Teller, BET, method) at 77 K using a TriStar II Plus system (Micromeritics, USA) after degassing for at least 3 h at 110 °C under a flow of nitrogen gas. XRD patterns were obtained at ambient temperature with a MiniFlex X-ray diffractometer (Rigaku Europe, Germany) using Cu Kα1 radiation (1.5406 Å) at 40 kV and 15 mA. The patterns were recorded between 10 and 80° 2θ at a step size of 0.01° 2θ and a scan speed of 2.00 deg min^–1^. A DTEX detector was used in narrow energy gap mode to suppress iron fluorescence background from the copper radiation. XRD data were analyzed using PDXL2 software (Rigaku Europe, Germany). All patterns were normalized relative to the intensity of the peak corresponding to the (311) crystal plane. The average crystal size (*d*_XRD_) of nanoparticles was calculated by Rietveld refinement analysis using the PDXL2 software. Particle morphology was visualized by TEM with a Tecnai G2 Spirit BioTWIN equipment (Thermo Fisher/FEI, USA) operating at 80 kV. The samples were suspended in 99.5% ethanol and deposited (as a 5 μL drop) on a 200 mesh copper grid (Ted Pella, USA) coated with formvar and carbon. The particle sizes were measured by counting 360 and 394 particles of Zn_0.5_Fe_2.5_O_4_ and Mn_0.5_Fe_2.5_O_4_, respectively, using ImageJ software. The particle sizes were plotted as histograms using the Sturges method^54^ and fitted to a log-normal distribution. The primary particle sizes were calculated as the geometric mean from the log-normal curve fitting. The particle magnetization was recorded on a vibrating sample magnetometer (Lake Shore, USA) and a Physical Property Measurement System (Quantum Design, USA). Magnetization versus magnetic field was measured in the field range ±1000 mT at a constant temperature of 25 °C. The thermal dissipation of nanoparticle powders was measured using an oscillating magnetic field apparatus (magneTherm; Nanotherics Ltd., UK). The heat dissipation from the bulk powders was measured with a fiber optic probe. Approximately 25 mg of each nanoparticle powder was transferred to a 2 mL glass vial and placed inside a coil with nine windings. The nominal oscillation frequency was set to 588.5 kHz and the magnetic field strength to 14 mT. The heating efficiency was calculated as the rise in temperature in the initial 10 s.

Cell viability assay was performed to assess the cytotoxicity of undoped and doped SPIONs. The cell culture media and reagents were purchased from Thermo Fisher Scientific (USA) or Sigma-Aldrich (USA). Caco-2 cells (originally obtained from the American Type Culture Collection), passage 95–105, were maintained in Dulbecco’s modified Eagle’s medium, containing 10% (v/v) fetal bovine serum and 1% (v/v) nonessential amino acids. The cells were cultured in a humidified incubator (at 37 °C, 10% CO_2_) in 75 cm^2^ tissue culture flasks. Stock suspensions of the nanoparticle powders in Milli-Q water were prepared at 10 mg mL^–1^ for γ-Fe_2_O_3_ and Mn_0.5_Fe_2.5_O_4_ and at 2 mg mL^–1^ for Zn_0.5_Fe_2.5_O_4_. The suspensions were prepared with a cup horn ultrasonicator (Sonics, USA) until no change in hydrodynamic diameter was observed. The hydrodynamic diameter was measured by dynamic light scattering (Litesizer 500, AntonPaar, Austria). The stock suspensions were diluted further with cell culture medium to achieve concentrations of nanoparticles at 100, 150, and 200 μg mL^–1^. Caco-2 cells were plated into black opaque 96-well plates at a density of 5 × 10^4^ cells per well in 300 μL of culture medium. The cells were allowed to attach for 24 h before treatment. Subsequently, the culture medium was replaced by 100 μL of particle suspensions in six replicates per treatment and incubated for 24 h (at 37 °C, 10% CO_2_). Positive controls were prepared by diluting 10% (w/v) sodium dodecyl sulfate (SDS) in water to achieve a final concentration of 0.22% (v/v) SDS in the cell culture medium. The culture medium was used as negative control. The water content in all treatments was kept at or below 2%. Finally, the viability of Caco-2 cells was evaluated with the CellTiter-Glo luminescent cell viability assay (Promega, USA). The luminescent signal from each well was determined with a plate reader (Tecan, Switzerland). A two-way analysis of variance (ANOVA) using Tukey’s multiple comparison test was used to compare the groups. Data analysis was performed using GraphPad Prism 9.0 software (La Jolla, CA, USA). *p* values were calculated as 0.1234 (ns), 0.0332 (*), 0.0021 (**), 0.0002 (***), and <0.0001 (****).

### Tablet Preparation and Hyperthermia-Induced Drug Amorphization

A DoE approach with a D-optimal design, resembling two, parallel central composite face (CCF; Figure S2)-centered fractional factorial designs, was used to study the effect of critical process parameters and their interactions on *T*_max_ and the degree of drug amorphization in the tablets. The experiment was set up using MODDE 12.1 (Umetrics AB, Sweden). The nanoparticle load was varied at 10, 15, and 20 wt % (with respect to the total tablet weight, Table S2) and the celecoxib load at 30, 40, and 50 wt % (with respect to the polymer weight, Table S2). Doped SPIONs (Zn_0.5_Fe_2.5_O_4_ and Mn_0.5_Fe_2.5_O_4_) and the duration of AMF exposure in solid state (3–15 min) were also included in the design. In total, 34 experiments were conducted (Table S3), of which each CCF design had eight full factorial points, three center points, and six star points. The models were fitted with multiple linear regression and adjusted by removing nonsignificant model terms. A value of *p* < 0.05 was considered significant. The surface contour plot was constructed to visualize the effect of the factors on tablet temperature and drug amorphization.

The tablets were prepared based on the experimental design. Physical mixtures of doped SPIONs, celecoxib (*M*_w_ = 381.4 g mol^–1^), PVP (*M*_w_ = 2000–3000 g mol^–1^), and magnesium stearate (0.5 wt %) (*M*_w_ = 591.3 g mol^–1^) were prepared using a mortar and pestle. Celecoxib and magnesium stearate were purchased from Fagron Nordic A/S (Denmark). Kollidon 12PF (PVP) was a kind gift from BASF (Germany). From the physical mixtures, 50 ± 2 mg flat-faced tablets (Ø 6 mm) were prepared using an instrumented single punch tablet press GTP-1 (Gamlen Instruments, UK). The tablet press was fitted with a 500 mg load cell (CT6-500-022) and used at a compression pressure of 160 MPa. The tablets and the mixtures were stored in airtight containers until further use.

The tablets were exposed to an AMF using magneTherm (Nanotherics Ltd., UK). They were placed on a glass Petri dish inside a coil with nine windings, and the nominal oscillation frequency was set to 588.5 kHz and the magnetic field intensity at 14 mT. The AMF exposure time was varied according to the DoE (Table S2). The increase in tablet surface temperature in the AMF was measured using an IR thermal camera (Fluke Ti480 Pro, Fluke Europe, the Netherlands). A thermal image was taken every third second for the first 3 min, and thereafter every 30 s. The IR images were analyzed using SmartView 4.3 (Fluke Europe, the Netherlands).

### Tablet Characterization

Tablets exposed to AMF were gently powderized by a mortar and pestle for subsequent solid-state analysis. The water content of the pure compounds, the physical mixtures, and the tablets after AMF exposure was determined with a thermogravimetric analyzer (Discovery, TA Instruments Ind., USA). The experiments were conducted under a nitrogen gas purge of 25 mL min^–1^ and a heating rate of 10 °C min^–1^ from ambient temperature to 170 °C. Each experiment was conducted in duplicate (*n* = 2) for the pure compounds and physical mixtures and as a single run for the powderized tablets. The weight loss (corresponding to the water content) was determined using the TA Instruments TRIOS software (version 5.1.1). Drug crystallinity of the pure substances, physical mixtures, and powderized AMF-exposed tablets was analyzed with an X-ray powder X’Pert Pro diffractometer (PANalytical, the Netherlands) using Cu Kα radiation (λ = 1.54187 Å). The diffractograms were recorded from 2θ 5 to 30° at 45 kV and 40 mA. As the superparamagnetic nanoparticles fluoresce, the pulse height distribution level was adjusted to 40–80% of the energy to compensate for the baseline drift and to obtain a higher signal-to-noise ratio. The diffractograms were analyzed using the X’Pert HighScore Plus software (version 2.2.4).

Differential scanning calorimetry (DSC; Discovery, TA Instruments, USA) scans were collected for the physical mixtures and the AMF-exposed tablets. All experiments were conducted under a nitrogen gas purge of 50 mL min^–1^. Using physical mixtures of 10–50 wt % celecoxib in PVP (in 10% increments), the melting enthalpy of the depressed melting was determined and fitted with a second degree polynomial function. The calibration experiments were conducted in duplicate (*n* = 2). Each sample (3–5 mg) was weighed in Tzero aluminum pans with hermetic lids. The lid was perforated to allow water evaporation. A modulated DSC run was performed at a heating rate of 3 °C min^–1^ from −20 to 170 °C after an isothermal period of 2 min. The modulation had an amplitude of 1 °C min^–1^ at a period of 50 s. The melting enthalpy was determined in the total heat flow signal. Each experiment was conducted once (*n* = 1). The sample mass and melting enthalpy were corrected for the water content determined by TGA. Using the calibration fitting, the crystallinity was calculated from the determined melting enthalpy. The relative residual crystallinity was calculated by dividing it with the crystallinity of celecoxib in the physical mixtures, that is, before exposure to the magnetic field. Degree of drug amorphization (%) is the noncrystalline fraction calculated from the relative residual crystallinity. The melting enthalpy was determined using the TRIOS software from TA Instruments (version 5.1.1).

The theoretical onset was determined by a DSC instrument (Q-2000, TA Instruments, New Castle, DE, USA), as described elsewhere.^55^ The onset temperature of the heating rates was extrapolated to a heating rate of 0 °C min^–1^. The extrapolated value corresponds to the respective onset temperature of the dissolution process for the three drug loadings of celecoxib in PVP. It should be noted that the determined temperatures correspond to water-free systems, and these will be lower for formulations that still contain small amounts of sorbed water due to the hygroscopicity of the polymer PVP.

High-performance liquid chromatography (HPLC) was conducted to quantify the amount of celecoxib in the physical mixtures and in the AMF-exposed tablets.^8^ In short, the experiments were performed using a 1260 Infinity HPLC system (Agilent Technologies, Inc., USA) on a reverse-phase Luna 5U C18(2) 100 A column (length, 150 mm; diameter 4.60 mm; Phenomenex Ltd., Germany). The experiments were performed at room temperature using a mobile phase consisting of 30% Milli-Q water and 70% ethanol (>99.7% HPLC grade; VWR). The flow rate was set at 1 mL min^–1^, and a sample volume of 20 μL was injected. UV detection was performed at 251 nm. Celecoxib eluted at about 2.7 min. The physical mixtures and the AMF-exposed tablets were dissolved in ethanol and filtered using a nylon syringe filter Q-max RR 25 mm with a pore size of 0.45 μm (Frisenette Aps, Denmark). Each sample was injected in triplicate (*n* = 3). The sample mass was corrected for the corresponding water content determined by TGA.

### In Vitro Dissolution of AMF-Exposed Tablets

Fasted state simulated intestinal fluid (FaSSIF) was used as a biorelevant medium to study the in vitro dissolution and was prepared according to the manufacturer’s instructions (Biorelevant.com). The mixture was equilibrated for 2 h before use and used within 48 h of preparation. Amorphous celecoxib and conventional ASD (30 wt % drug load) were prepared by the melt quenching technique.^56^ The drug–polymer ratio for the conventional ASD was selected based on the tablet, resulting in the maximum amorphous content induced by in situ amorphization (tablet 26, Table S3). For the ASD, the drug and polymer were weighed and mixed thoroughly using a mortar and pestle. This mixture was spread in a thin layer on a sheet of aluminum foil and placed in an oven at 169 °C for 5 min, after which it was removed, cooled to room temperature, and pulverized using a mortar and pestle. This process was repeated twice for the ASD and once for amorphous celecoxib.

The nonsink dissolution measurements were performed in FaSSIF using the μDISS Profiler (Pion Inc., USA).^57^ Each channel was calibrated with its own standard curve prior to the experiment. For calibration, stock solutions were prepared using dimethyl sulfoxide containing either crystalline celecoxib alone or celecoxib (30 wt %) in PVP. The standard curves were established with 6–8 concentrations by sequentially adding 5 μL aliquots of the stock solution into 3 mL of FaSSIF maintained at 37 ± 1 °C and stirred at 800 rpm. The standard curve showed a linear correlation *R*^2^ ≥ 0.99 over a range of 15–270 μg mL^–1^. The dissolution and solubility of all compounds were determined in FaSSIF. The path lengths (2–20 mm) of the in situ UV probes were selected based on the drug solubility in the selected medium and the expected degree of supersaturation. When a high solubility was anticipated, the shorter probe length (5 mm) was used. The medium was preheated to 37 °C prior to use in the dissolution experiment. The measurement was initiated by adding an excess of drug to the vials, followed by 15 mL of preheated FaSSIF. A dose of 400 μg mL^–1^ of celecoxib was selected based on the recommended minimum dosage for rheumatoid arthritis and the intestinal fluid volume.^58,59^ Vials containing crystalline and amorphous celecoxib, and ASD (30 wt % drug load), were stirred using cross-bar magnetic stirrers at a speed of 200 rpm, whereas the vials with Mn ferrite containing powders were shaken at 300 rpm to avoid interaction with the magnetic stir bar. All samples were maintained at 37 ± 1 °C and analyzed at 1, 5, 10, 15, 20, 25, 30, 45, 60, 120, 180, 240, and 360 min with the UV probes. Each experiment was run in triplicates except the AMF-exposed Mn ferrite-containing tablets, which were run in duplicates. The second derivative spectra were used to determine the concentration of celecoxib in the medium over time. *C*_max_ and the time to reach *C*_max_ (*t*_max_) were obtained by noncompartmental analysis of the in vitro dissolution data. ANOVA using Tukey’s multiple comparison test was used to compare the *C*_max_ values of various formulations. Data analysis was performed using GraphPad Prism 9.0 software (La Jolla, CA, USA). *p* values were calculated as 0.1234 (ns), 0.0332 (*), 0.0021 (**), 0.0002 (***), and <0.0001 (****).

## Supporting Information Available

Results from the formulation design space, summary of DoE, modeling of amorphization, correlation of degree of drug amorphization and *T*_max_, and in vitro dissolution assays (PDF)

## Abbreviations

SPION: superparamagnetic iron oxide nanoparticle
AMF: alternating magnetic field
ASD: amorphous solid dispersion
PVP: polyvinylpyrrolidone
FSP: flame spray pyrolysis
DoE: design of experiments
XRD: X-ray diffraction
TEM: transmission electron microscopy
DSC: differential scanning calorimetry
SDS: sodium dodecyl sulfate
FaSSIF: fasted state simulated intestinal fluid
TGA: thermogravimetric analysis
HPLC: high-performance liquid chromatography
