# Supplementary material for: Hyperthermia-Induced In Situ Drug Amorphization by Superparamagnetic Nanoparticles in Oral Dosage Forms
Source: ACS Appl Mater Interfaces. 2022 Apr 22;14(19):21978–88. doi: 10.1021/acsami.2c03556 (PMC9121342; doi:10.1021/acsami.2c03556)
Supplement: Supplementary file 1 — am2c03556_si_001.pdf [file am2c03556_si_001.pdf]

Supporting Information

Hyperthermia-induced in situ drug amorphization by superparamagnetic nanoparticles in oral dosage forms

*Shaquib Rahman Ansari[a], Nele-Johanna Hempel[b§], Shno Asad[a], Peter Svedlindh[c], Christel A. S. Bergström[d], Korbinian Löbmann[b], and Alexandra Teleki[a]**

[a] Department of Pharmacy, Science for Life Laboratory, Uppsala University, 75123 Uppsala, Sweden

[b] Department of Pharmacy, University of Copenhagen, 2100 Copenhagen, Denmark

[c] Department of Materials Science and Engineering, Uppsala University, 75103 Uppsala, Sweden

[d] Department of Pharmacy, Uppsala University, 75123 Uppsala, Sweden

*Email: alexandra.teleki@scilifelab.uu.se

Table S1. Lattice constant (*a*), average particle size from XRD (d_{XRD}) and TEM (d_{TEM}), specific surface area (SSA), saturation magnetization (M_s) and heating efficiency of the three SPION types.

**SPION type**	***a*** **[Å]**	**d_{XRD} [nm]**	**d_{TEM} [nm]**	**SSA [m^2 g^{-1}]**	**M_s [emu g^{-1}]**	**ΔT after 10 sec [°C]**
γ-Fe_2O_3	8.3424	14.6	-	81.2	53.7	61.6
$Zn_{0.5}Fe_{2.5}O_4$	8.4023	13.7	17.6	64.3	53	79.6
$Mn_{0.5}Fe_{2.5}O_4$	8.4167	18.1	17.3	52.3	66.7	164.3

Table S2. Formulation design space showing the factors investigated in the hyperthermia-induced drug amorphization.

**Factor**	**Abbreviation**	**Levels**
SPION composition	-	$Zn_{0.5}Fe_{2.5}O_4$, $Mn_{0.5}Fe_{2.5}O_4$
Doped SPION content in tablets [wt%]	SPION content	10, 15, 20
Drug load with respect to polymer [wt%]	Drug load	30, 40, 50
AMF exposure of tablets [min]	AMF time	3, 9, 15

Table S3. Summary of design of experiments (DoE).

Experiment number	SPION composition	SPION content [wt%]	Drug Load [wt%]	AMF time [min]	T_{max} [°C]	Degree of amorphization [%]
1	$Zn_{0.5}Fe_{2.5}O_4$	10	30	15	67.9	7.7
2	$Zn_{0.5}Fe_{2.5}O_4$	10	40	9	79.9	0.1
3	$Zn_{0.5}Fe_{2.5}O_4$	10	30	3	67.5	18.0
4	$Zn_{0.5}Fe_{2.5}O_4$	10	50	3	77.7	7.6
5	$Zn_{0.5}Fe_{2.5}O_4$	10	50	15	85.3	7.0
6	$Zn_{0.5}Fe_{2.5}O_4$	15	30	9	93.2	22.6
7	$Zn_{0.5}Fe_{2.5}O_4$	15	40	3	94.8	13.8
8	$Zn_{0.5}Fe_{2.5}O_4$	15	40	9	92.9	17.3
9	$Zn_{0.5}Fe_{2.5}O_4$	15	40	9	86.1	8.8
10	$Zn_{0.5}Fe_{2.5}O_4$	15	40	9	100.1	23.4
11	$Zn_{0.5}Fe_{2.5}O_4$	15	40	15	107	30.9
12	$Zn_{0.5}Fe_{2.5}O_4$	15	50	9	80.3	10.5
13	$Zn_{0.5}Fe_{2.5}O_4$	20	30	3	98.6	20.5
14	$Zn_{0.5}Fe_{2.5}O_4$	20	30	15	127.3	52.5
15	$Zn_{0.5}Fe_{2.5}O_4$	20	40	9	106.4	36.6
16	$Zn_{0.5}Fe_{2.5}O_4$	20	50	3	108	24.9
17	$Zn_{0.5}Fe_{2.5}O_4$	20	50	15	107.9	19.9
18	$Mn_{0.5}Fe_{2.5}O_4$	10	30	3	110.3	47.4
19	$Mn_{0.5}Fe_{2.5}O_4$	10	30	15	90.5	20.3
20	$Mn_{0.5}Fe_{2.5}O_4$	10	50	3	92	6.5
21	$Mn_{0.5}Fe_{2.5}O_4$	10	50	15	99.8	20.8
22	$Mn_{0.5}Fe_{2.5}O_4$	15	40	9	127.6	64.1
23	$Mn_{0.5}Fe_{2.5}O_4$	15	40	9	147.1	76.9
24	$Mn_{0.5}Fe_{2.5}O_4$	15	40	9	128.1	62.3
25	$Mn_{0.5}Fe_{2.5}O_4$	20	30	3	143.1	83.9
26	$Mn_{0.5}Fe_{2.5}O_4$	20	30	15	165.2	100
27	$Mn_{0.5}Fe_{2.5}O_4$	20	50	3	154.9	78.6
28	$Mn_{0.5}Fe_{2.5}O_4$	20	50	15	152.1	83.9
29	$Mn_{0.5}Fe_{2.5}O_4$	15	30	9	145	78.9
30	$Mn_{0.5}Fe_{2.5}O_4$	10	40	9	112	49.2
31	$Mn_{0.5}Fe_{2.5}O_4$	15	50	9	142.5	69.9
32[a)]	*$Mn_{0.5}Fe_{2.5}O_4$*	*15*	*40*	*15*	*111.9*	*18.5*
33	$Mn_{0.5}Fe_{2.5}O_4$	15	40	3	132.3	54.4
34	$Mn_{0.5}Fe_{2.5}O_4$	20	40	9	164	86.3

[a)]*Italics*: Experiment number 32 was considered an outlier and removed from the final regression model.

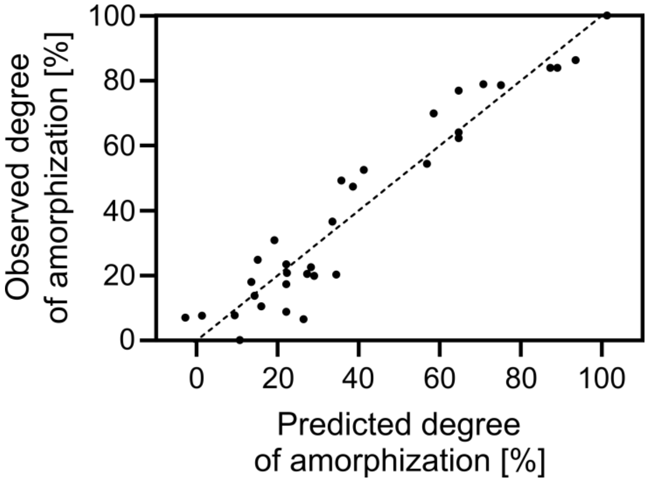

Figure S1. Correlation between the measured and the predicted degree of amorphization after establishment of the model. The dashed line represents linear fit.

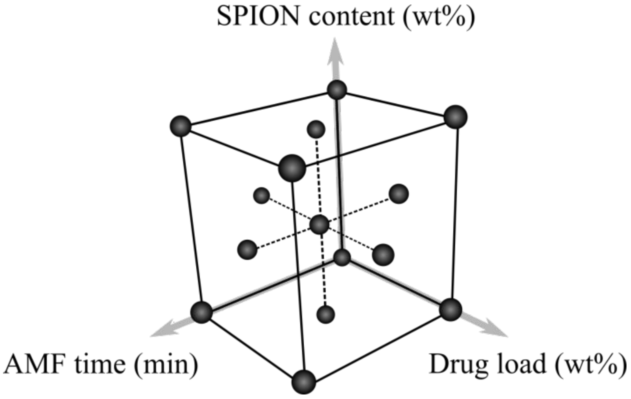

Figure S2. Design region of the two parallel central composite face (CCF) centered model. Identical designs were used for both SPION compositions.

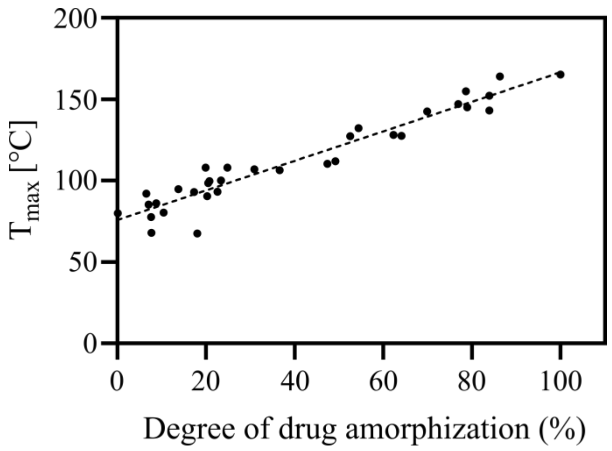

Figure S3. Correlation between the degree of drug amorphization and maximum tablet temperature (T_{max}). The dashed line represents linear fit. (r = 0.96)

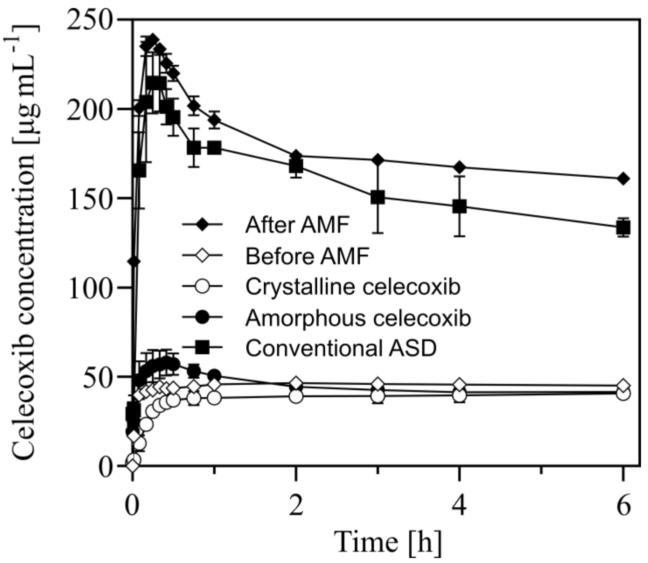

Figure S4. *In vitro* dissolution profiles (up to 6 hours) of tablets at a dose corresponding to 400 µg mL^{-1} of celecoxib in simulated intestinal fluid (FaSSIF). Crystalline celecoxib (open circles); amorphous celecoxib (filled circles); conventional amorphous solid dispersion of celecoxib (30 wt%) in PVP (filled squares); tablet containing $Mn_{0.5}Fe_{2.5}O_4$ (20 wt%) and celecoxib (30 wt%) in PVP before AMF exposure (open diamonds) and after AMF exposure of 15 min (filled diamonds). Values represent mean celecoxib concentrations ± SD, n = 3 (n = 2 for tablet containing Mn ferrites after AMF exposure).
